# Esophageal perforation after perioperative transesophageal echocardiography: a case report

**DOI:** 10.1186/s13256-016-1131-0

**Published:** 2016-12-01

**Authors:** Hyun-Chang Kim, Jung-Hyou Oh, Yong-Cheol Lee

**Affiliations:** Department of Anesthesiology and Pain Medicine, Keimyung University School of Medicine, 56, Dalseong-ro, Jung-gu, Daegu 41931 Korea

**Keywords:** Transesophageal echocardiography, Complications, Esophageal perforation, Postoperative period, Case report

## Abstract

**Background:**

Transesophageal echocardiography is widely used in cardiac surgery. Transesophageal echocardiography probe insertion and manipulation can injure the esophagus.

**Case presentation:**

A 76-year-old Asian man was admitted to our hospital for coronary artery bypass graft revascularization surgery. A right carotid endarterectomy was successfully performed 2 days before coronary artery bypass graft revascularization surgery. After the coronary artery bypass graft revascularization surgery was done successfully, postoperative computed tomography and esophagography revealed perforation of the middle esophagus. The patient was managed with fasting, parenteral nutrition, and intravenous antibiotics.

**Conclusions:**

Esophageal perforation can occur with transesophageal echocardiography probe insertion, even without difficulty or resistance, in patients with atherosclerotic disease. Appropriate imaging and vigilance for esophageal injury after a transesophageal echocardiography examination in patients with cardiovascular disease are necessary for appropriate diagnosis and treatment.

## Background

Transesophageal echocardiography (TEE) is a standard intraoperative monitoring tool for patients undergoing cardiac surgery. Although TEE is considered relatively safe, it may result in complications [1]. The insertion, manipulation, and removal of the TEE probe during cardiac surgery may increase complications. Among these complications, esophageal perforation is rare but life-threatening [2]. We present a case of a patient with esophageal perforation after perioperative TEE.

## Case presentation

A 76-year-old, 166-cm, 71.3-kg Asian man with chest pain of 2 months’ duration due to coronary artery disease was admitted for coronary artery bypass graft (CABG) revascularization surgery. He had a history of hypertension and diabetes mellitus. He had a dilated left atrium, minimal tricuspid regurgitation, and normal left ventricular systolic function. He had no history of dysphagia or esophageal regurgitation. Preoperative magnetic resonance angiography revealed that his right internal carotid artery was severely occluded. The neurologist recommended a right carotid endarterectomy before CABG. The right carotid endarterectomy was performed uneventfully 2 days before CABG, and the total anesthesia time was 3 h 30 minutes.

A peripheral intravenous catheter was inserted into the left forearm, and an arterial catheter was inserted at the right radial artery. Anesthesia was induced with remifentanil 32 μg, propofol 120 mg, and rocuronium 70 mg. We inserted an OmniPlane TEE probe (Philips Medical Systems, Andover, MA, USA) into the esophagus without any difficulty or resistance. TEE examination was done with the CABG procedure. For patient safety, the comprehensive intraoperative TEE guidelines of the American Society of Echocardiography Council for Intraoperative Echocardiography and the Society of Cardiovascular Anesthesiologists Task Force were followed.

The CABG was successful. The cardiopulmonary bypass time was 105 minutes. During cardiopulmonary bypass, the patient’s temperature was lowered to 33.0 °C at the rectum (33.0 °C at the nasal membrane). The TEE probe was in a neutral position in the upper esophagus and frozen to prevent injury. The average pump flow rate was 2.4 L/minute/m^2^. At the end of the procedure, the patient was rewarmed to 36.3 °C at the rectum (36.8 °C at the nasal membrane) for 60 minutes. During rewarming, TEE examination was performed, and the temperature of the TEE probe was increased to 39 °C. The TEE examination was discontinued intermittently and automatically to reduce the probe temperature. During the TEE examination, any movement or manipulation was gentle. Dopamine and nitroglycerin were given during weaning from bypass. The TEE probe was removed at the end of anesthesia. It was clean with no signs of blood. Extubation was done 11 h postoperatively.

**Fig. 1:**
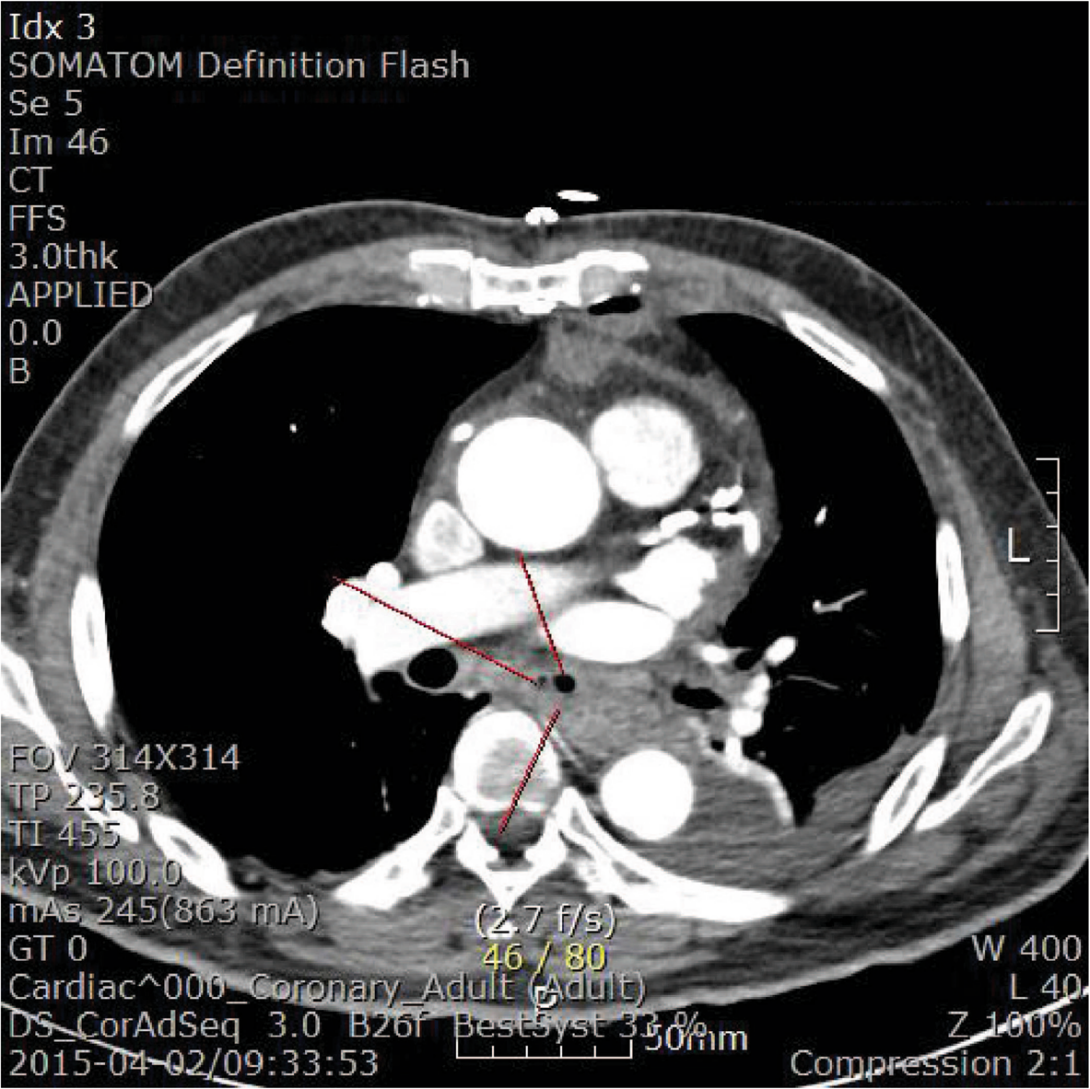
Chest computed tomographic scan. Abscess formation or air-containing tissue in retrosternal, pericardial, and paraesophageal areas is seen. Red lines indicate esophageal wall thickening

**Fig. 2:**
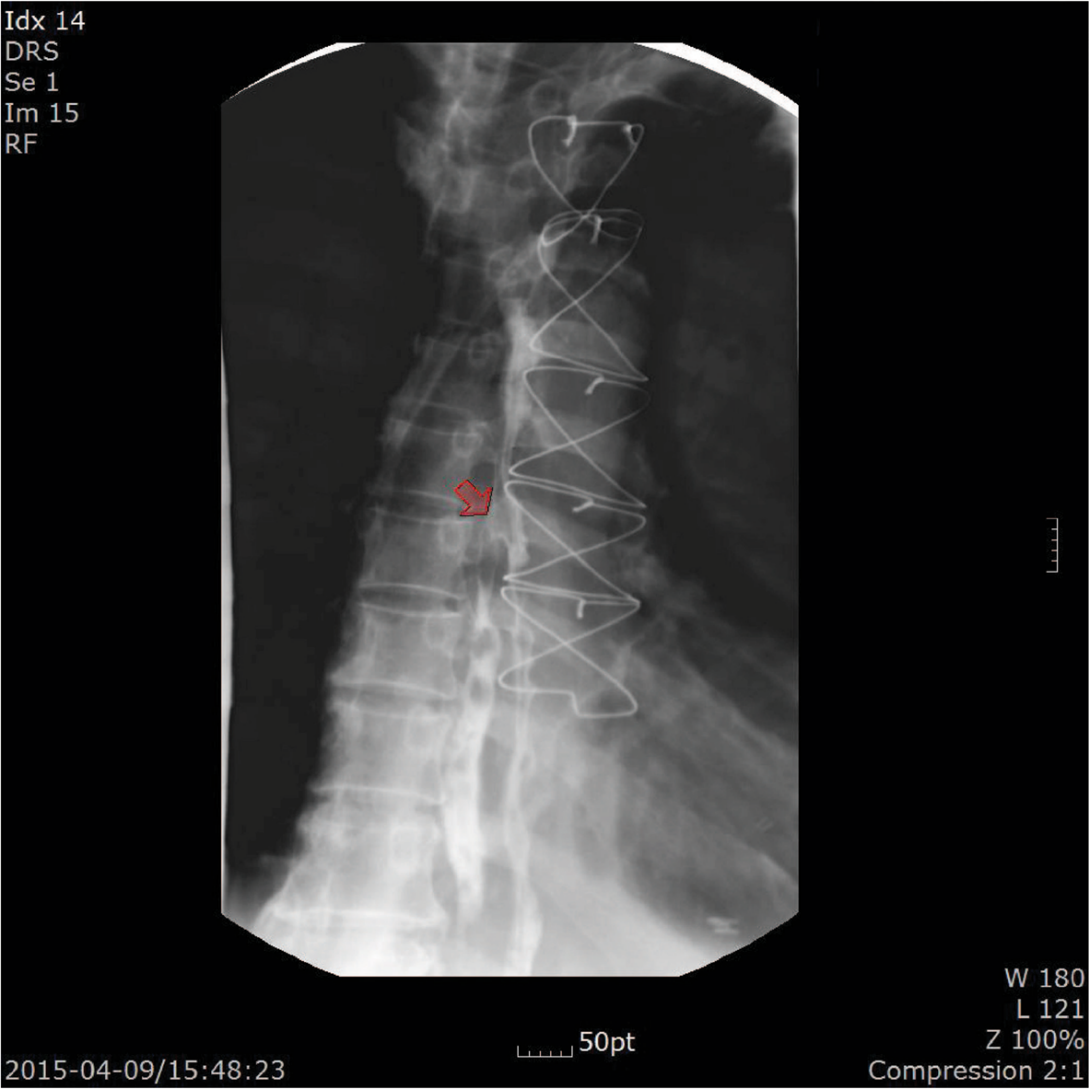
Esophagography. A contrast leakage in the right wall of the middle esophagus is visible (*arrow*)

The patient developed intermittent fever on the first postoperative day. Leukocytosis (19,440 white blood cells/μl) and a high level of C-reactive protein (30.0 mg/dl) were detected in the postoperative laboratory examination. The patient’s fever ranged from 37.0 °C to 38.3 °C and did not subside until postoperative day 3, despite empirical treatment with tazobactam and ciprofloxacin. Computed tomography done to evaluate the coronary arteries incidentally found signs of abscess formation or air-containing tissue in the retrosternal, pericardial, and paraesophageal areas (Fig. 1). Esophagography revealed contrast leakage in the right wall of the middle esophagus (Fig. 2). The patient’s hemodynamic variables, including blood pressure and heart rate, remained stable and were monitored closely. The patient was managed conservatively with fasting, parenteral nutrition, and intravenous antibiotics. We were prepared for surgical intervention, such as open esophageal repair or endoscopic primary repair, if there was any indication of unstable vital signs. The patient’s postoperative fever abated on postoperative day 4. Follow-up esophagography disclosed no change in the esophageal lesion. The patient was allowed to drink sips of water 35 days postoperatively. On postoperative day 39, he began a normal diet. He was discharged without complications on postoperative day 42.

## Discussion

TEE is used widely during cardiac surgery. Based on the American Society of Anesthesiologists and the Society of Cardiovascular Anesthesiologists Task Force practice guidelines, the use of TEE in our patient was strongly indicated [3]. TEE is considered as a safe procedure, but it may result in serious complications, such as esophageal injury, vocal cord paralysis, arrhythmia, hypotension, seizure, and cardiac arrest [4]. An upper gastrointestinal tract injury is a rare, devastating complication of TEE. The incidence of iatrogenic esophageal perforation by TEE is 0.03–0.09% [5, 6]. The incidence of thoracic esophageal perforation during TEE examination is higher in the intraoperative setting than in a nonoperative setting [7].

**Fig. 3:**
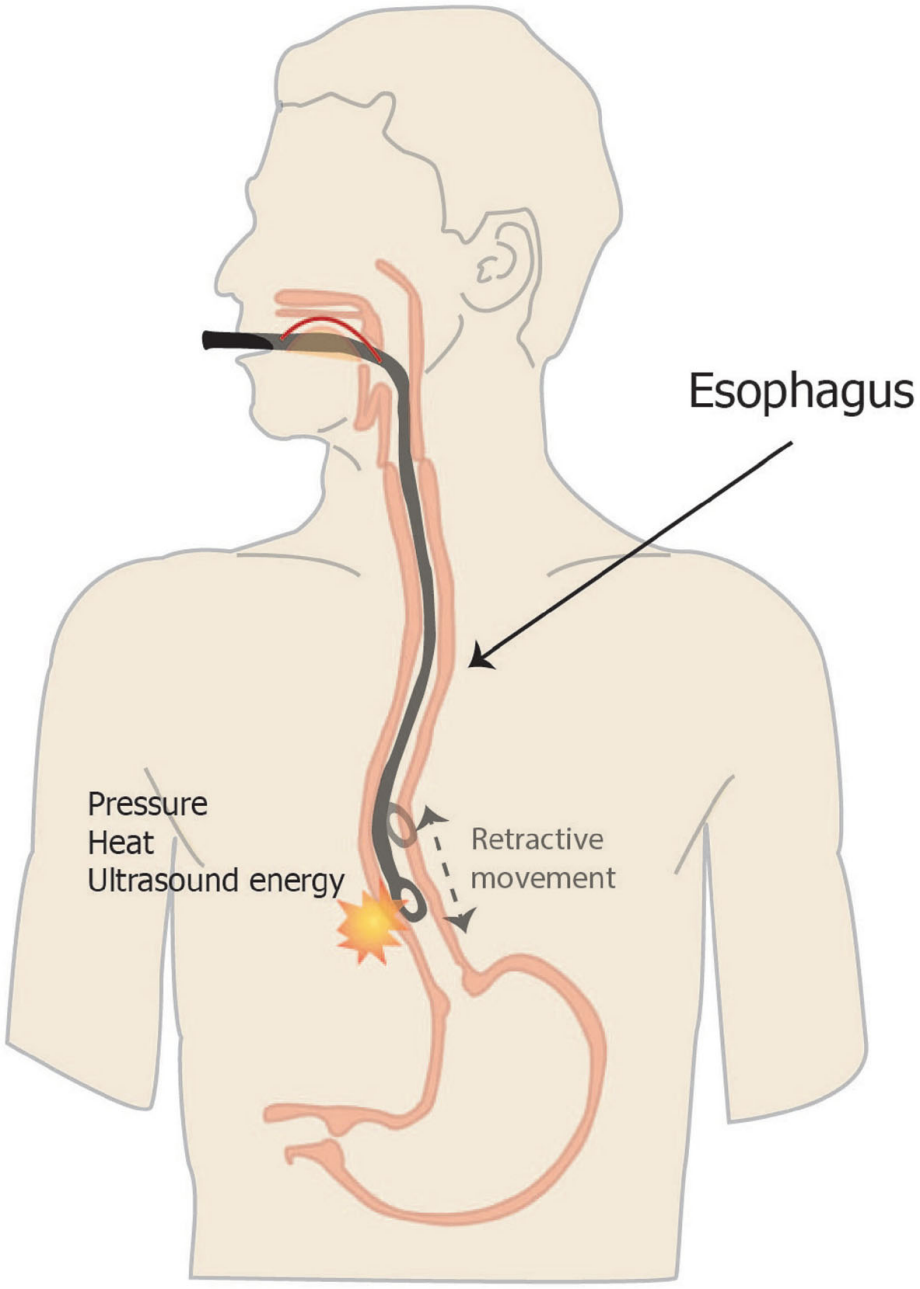
Schematic diagram of mechanisms of esophageal perforation by transesophageal echocardiographic probe

There are various mechanisms for esophageal injury caused by TEE. A blind insertion, advancement, flexion, or manipulation of the TEE probe may result in mechanical trauma to the upper gastrointestinal tract [8]. Prolonged, continuous pressure by the TEE probe may reduce blood supply at the esophagus and cause necrosis [9]. The heat or ultrasound energy produced by the TEE probe may contribute to esophageal injury [7]. In our patient, the distal thoracic segment of the esophagus was injured. The TEE probe was retracted into the upper esophagus after each examination to prevent gastrointestinal injury. This repeated maneuvering of the probe may paradoxically increase the risk of esophageal perforation (Fig. 3). A high probe temperature during rewarming may also result in esophageal perforation. Avoiding unnecessary movement of the TEE probe and keeping the TEE probe normothermic after the examination is recommended to prevent esophageal injury.

Our patient did not have common risk factors for esophageal injury, such as a history of dysphagia, esophageal disease, chest radiation, large left atrium, chronic steroid treatment, cervical vertebrae osteophytic change, or resistance during TEE insertion. Our patient did have severe atherosclerotic vascular disease, which required a carotid endarterectomy before CABG. The circulation of the esophagus may be compromised in patients with atherosclerotic disease [10].

The mortality of gastrointestinal perforation ranges from 10% to 56% [11, 12]. Delayed detection of esophageal perforation may lead to mediastinitis, severe sepsis, and death. In patients who are sedated after cardiac surgery, it is nearly impossible to detect the signs of esophageal injury, such as vomiting and pain. Chest radiographs often appear normal in patients with esophageal injury [13]. In our patient, postoperative chest radiographs did not show any sign of esophageal perforation, including pneumomediastinum. The patient’s nonspecific postoperative fever was the only objective sign of esophageal injury. Chest computed tomography for postoperative coronary artery evaluation revealed the esophageal injury, and subsequent esophagography confirmed the diagnosis. We believe that adequate conservative management with fasting enabled our patient to survive without complications. We recommend a high level of suspicion for gastrointestinal tract injury and meticulous evaluation of any postoperative imaging studies in patients with any abnormal findings such as a fever after a cardiac revascularization procedure requiring TEE insertion.

## Conclusions

Esophageal perforation can occur with TEE probe insertion when there is no difficulty or resistance in patients with atherosclerotic disease. Therefore, one must always consider the possibility of gastrointestinal injury after a TEE examination. Appropriate imaging study and a high level of vigilance for esophageal injury after a TEE examination in patients with cardiovascular disease are necessary for appropriate diagnosis and therapy. TEE procedures need to be done carefully, especially in atherosclerotic patients. Careful identification of risk factors and gentle manipulation of the TEE probe may prevent life-threatening complications after a perioperative TEE examination.

## Abbreviations

CABG: Coronary artery bypass graft
TEE: Transesophageal echocardiography
